# High transmission risk of Human Immunodeficiency Virus in blood transfusions: A critical evaluation of blood bank serological tests in Mozambique

**DOI:** 10.1371/journal.pgph.0007398

**Published:** 2026-09-30

**Authors:** Nédio Mabunda, Odete Matola, Júlia Sambo, Imelda Chelene, Abras Munguambe, Nuro Abílio, Adolfo Vubil, Osvaldo Loquiha, Ilesh Jani

**Affiliations:** 1 Instituto Nacional de Saúde, Marracuene, Mozambique; 2 Clinton Health Access Initiative, Maputo city, Mozambique; 3 Universidade Eduardo Mondlane, Maputo city, Mozambique; PLOS: Public Library of Science, UNITED STATES OF AMERICA

## Abstract

Many settings in sub-Saharan Africa use rapid antibody assays to detect human immunodeficiency virus (HIV) infection among blood donor candidates. This study aimed to evaluate the analytical performance of HIV serological tests used to screen blood donor candidates in Mozambique. Determine HIV 1/2 + Uni-Gold HIV (the routine HIV testing at blood banks in Mozambique), and two newer rapid tests (Determine HIV Early Detect and Meriscreen HIV1–2 Western Blot) were compared with an Enzyme-Linked Immunosorbent Assays (Merilisa HIV Gen 4 test + Uni-Gold HIV). Additionally, all serological tests were compared with the molecular cobas MPX test. The sensitivity and specificity of Determine HIV 1/2 + Uni-Gold HIV; Determine HIV Early Detect, and Meriscreen HIV1–2 Western Blot compared to the Merilisa HIV Gen 4 test + Uni-Gold were 85.5% and 99.6%; 88.7% and 99.5%; 90.0% and 99.6% respectively. The sensitivity and specificity of Determine HIV 1/2 + Uni-Gold HIV; Determine HIV Early Detect, Meriscreen HIV1–2 Western Blot, and Merilisa HIV Gen 4 test + Uni-Gold compared to the cobas MPX test were 64.0% and 99.1%; 68.6% and 99.1%; 70.8% and 99.0%; 66.0% and 99.4%, respectively. The probability of undetected positive results in the rapid tests and Merilisa HIV Gen 4 test ranged from 5.6 to 6.7 per 1,000 blood donors. The results of this study point to the need for the introduction of molecular testing for HIV in blood banks in developing countries to improve transfusion safety and contribute to controlling HIV as a public health problem.

## Introduction

Blood transfusion is an important transmission route for the human immunodeficiency virus (HIV), hepatitis B virus (HBV), hepatitis C virus (HCV) and *Treponema pallidum* [1], and testing for blood-borne pathogens is recommended before any transfusion [2,3]. Advancements in diagnostic tests have contributed to substantially reduce the risk of transfusion-transmitted infections (TTIs) [4]. Importantly, nucleic acid tests (NAT), used alone or in conjunction with serological assays, have decreased the pathogen diagnostic window period to a few days only [5,6].

Sub-Saharan Africa, where HIV is highly prevalent, has lagged behind in modernizing transfusion services, including its testing infrastructure and equipment [7,8]. Due to limited resources, screening for HIV is performed using rapid diagnostic tests (RDTs) to detect antibodies in most blood banks in sub-Saharan Africa [9]. However, RDTs have a larger diagnostic window than instrument-based assays such as Enzyme-Linked Immunosorbent Assays (ELISA) and NAT [10].

Despite their relatively low analytical sensitivity, antibody RDTs are simple to perform, require no additional equipment or sophisticated infrastructure, and provide results within minutes [11]. The fourth generation RDTs, that detect HIV antibodies as well as the p24 antigen, and antibody rapid assays with improved designs became available with the promise of reducing the current diagnostic window [12].

The testing for TTIs (HIV, HBV, HCV, and *Treponema pallidum*) in Mozambique relies predominantly on RDTs with only 2.8% (2/172) of transfusion sites utilizing ELISA tests. All samples were screened for HIV following a sequential algorithm of two rapid antibody tests (Determine HIV 1/2 and Uni-Gold HIV) or ELISA tests and RDT (Merilisa HIV Gen 4 test and Uni-Gold HIV) [13].

Previous work in Mozambique showed that approximately three HIV-positive units per 1,000 donated blood units were misclassified as negative when an ELISA algorithm with rapid test confirmation was used [14].

To better understand this gap, this study evaluated the analytical performance of different HIV rapid tests (Determine HIV 1/2 and Uni-Gold HIV, Determine HIV Early Detect test, and the Meriscreen HIV1–2 WB test) for screening blood transfusion donors in areas with high HIV prevalence.

## Materials and methods

### Ethics statement

This study was approved by the Mozambique National Health Bioethics Committee, on November 9, 2022, with reference 753/CNBS/22. All procedures were carried out following the Helsinki Declaration as revised in 2013. Written informed consent was obtained from all blood donors before enrolment in the study.

### Study design and setting

This cross-sectional study was carried out in Gaza and Inhambane provinces in southern Mozambique. Eight health facilities, five in Gaza and three in Inhambane, participated in the study. Health facilities were selected by convenience, based on their high volume of blood donations.

Gaza province has an estimated population of 1,488,103 [15], and an HIV prevalence of 20.9% among 15–49 years olds, the highest in the country [16]. The average number of blood donations per year in the province is 5,800 and the sites chosen for the study contribute approximately 70% of the annual blood donations [13]. Inhambane province has an estimated population of 1,598,383 [15], and an HIV prevalence of 12.6% [16]. The annual average number of blood donations is estimated at 2,200, with study sites accounting for 60% of these [13]. Blood samples from these sites are demographically and clinically comparable to those from lower-volume sites in Mozambique.

### Study participants

Individuals aged 18–65 years who consecutively presented for blood donation at these health facilities were enrolled in the study. Recruitment was conducted during routine interviews used to assess donor eligibility. Individuals presenting severe illness, cognitive impairment, or altered mental status that prevented them from understanding the study procedures, providing informed consent, or completing the study interview were excluded. In addition, individuals who reported being permanently deferred from blood donation in Mozambique were also excluded.

### Sample size calculation

The study was designed to estimate sensitivity with a desired precision. Based on an expected sensitivity of 90% for the reference assays, a margin of error of 5%, and an anticipated HIV seroprevalence of 4% among blood donors, we calculated that a minimum sample size of 3,457 participants would be required.

### Data and samples collection

Demographic data and samples were collected between December 20, 2022, and February 14, 2024. Study participants’ information, including age, sex, donor type, and other characteristics, was obtained from consenting blood donors using a structured questionnaire (S1 Text). Three 6 mL K2EDTA vacuum tubes (Becton Dickinson, Franklin Lakes, NJ, USA) were collected from each consenting participant by the technician responsible for assessing the candidate’s health status before blood donation.

The samples were sent to the local laboratory, where plasma was obtained from whole blood and stored in three cryovials. One of the cryovials was used for on-site testing, while others were preserved at -20ºC for serological and molecular tests to be performed at the Maputo Central Hospital (HCM) and Instituto Nacional de Saúde of Mozambique (INS), respectively. Plasma samples were transported monthly to the testing laboratory (INS) and stored at -20°C until the day of testing. Remaining samples were stored at -80°C.

### HIV testing

#### Serological tests.

All samples were first tested for HIV at the blood bank according to a sequential algorithm of two RDTs (Details of the serological tests can be found in the supplementary material- S1 Table) as per national guidelines [17]. Briefly initial testing was performed using Determine HIV 1/2 (Abbott Rapid Diagnostics, Japan), and reactive samples were confirmed using Uni-Gold HIV (Trinity Biotech Plc, Ireland). Samples were considered negative if non-reactive on the initial test, and positive if reactive on both initial and confirmatory tests. A reactive result on Determine HIV 1/2 and a non-reactive result on Uni-Gold HIV was considered indeterminate. This procedure used blood samples collected routinely for this purpose.

Additionally, as part of the study procedures, samples were tested onsite using the Determine HIV Early Detect test, and the Meriscreen HIV1–2 WB test. The results of both tests were interpreted according to the manufacturer’s instructions.

Technicians at all blood banks were trained to perform the new tests and were updated on routine tests before study inititiation. Remote monitoring was performed via the online database Open Data Kit – ODK (www.getodk.org), complemented by monthly on-site visits during the recruitment phase.

The Merilisa HIV Gen 4 test (Meril Diagnostics, India), a qualitative enzyme immunoassay, was performed remotely at the Blood Bank of Maputo Central Hospital (HCM). The HCM blood bank serves as the national reference blood bank and uses ELISA methods to routinely screen for TTIs. The test was performed according to the manufacturer’s instructions. Samples with reactive results on Merilisa HIV Gen 4 were confirmed using Uni-Gold HIV. Samples were considered negative if non-reactive on Merilisa Gen 4 and positive if reactive on both the initial and confirmatory tests. A reactive result on Merilisa HIV Gen 4 followed by a non-reactive result on Uni-Gold HIV was considered indeterminate.

The technicians who performed HIV testing using ELISA did so blinded (without the results of rapid tests and NAT).

#### Qualitative HIV RNA detection.

HIV RNA was detected using the cobas MPX test (Roche Molecular Systems, Inc., Branchburg, NJ, USA), using the cobas 6800 systems with 850 µL of plasma per sample. The cobas MPX test enables simultaneous detection and differentiation of HIV RNA, HCV RNA, HBV DNA and the internal control (IC) in a single test. Detection limits are set at 25 IU/mL, 7 IU/mL and 1.4 IU/mL for HIV-1, HCV and HBV, respectively [18]. Cobas MPX has a sensitivity and specificity of 100% for HIV testing [19].

The technicians who performed HIV testing using NAT did so blinded (without the results of rapid tests and ELISA).

#### Quality control of laboratory testing.

Repeat serological and molecular testing for HIV took place at the Serology and Biotechnology and Genetics laboratories of INS. Both laboratories are national references for serological and molecular HIV testing in Mozambique and are accredited by IPAC (Portuguese Accreditation Institute) according to ISO 15189: 2012.

The HCM Blood Bank is a national reference blood bank. For HIV testing using the Merilisa HIV Gen 4 test, 5% of samples tested per month were randomly selected and retested at the INS Serology Laboratory.

All rapid test results (national algorithm and new rapid tests) that disagreed with the Merilisa HIV Gen 4 test were retested in duplicate at the serology laboratory.

### Statistical analysis

Frequency tables were constructed for categorical variables to provide a comprehensive description of socio-demographic characteristics of the study population. In addition, an estimate of positivity rate of each diagnostic test was determined and presented by age, gender and donor type.

Sensitivity, specificity, positive predictive value (PPV), negative predictive value (NPV), and agreement measures, including Cohen’s kappa coefficient and positive and negative agreement, were calculated to assess the performance of each RDT using Merilisa HIV Gen 4 and cobas MPX as reference standards.

For each comparison between a rapid test and a reference standard, we performed a complete-case analysis. Only samples with available results for both the specific rapid test and the reference standard under evaluation were included in the corresponding 2x2 table and performance calculations.

The false omission rate (FOR) was also calculated to estimate the proportion of potentially infected blood samples that were not identified by diagnostic tests. FOR is the proportion of positive cases that are incorrectly classified as negative by the diagnostic test. The FOR was multiplied by 1,000 to estimate the number of potentially infected blood donations per 1,000 donors screened. Error bars represent 95% confidence intervals calculated using the binomial approximation. Pairwise comparisons of FOR between assays were performed using two‑sample tests for equality of proportions.

Inferential statistics were employed to estimate the precision of the calculated measures. A 95% confidence interval was calculated for sensitivity, specificity, PPV, NPV and FOR to provide a range of plausible values for these indicators. All 95% confidence intervals for proportions (sensitivity, specificity, positive predictive value, negative predictive value, and false omission rate) were calculated using the normal approximation to the binomial distribution.

Indeterminate results on both the reference standard and the rapid tests were excluded from the 2x2 analyses, as they do not represent a definitive positive or negative classification.

The statistical analysis was produced using RStudio/2022.12.0 + 353 (R Foundation for Statistical Computing) and Microsoft Excel 2018.

## Results

### Characteristics of the study population

A total of 3,854 blood donor candidates were enrolled in the study, 2,390 (62.0%) from Gaza province and 1,464 (38.0%) from Inhambane province (Table 1). The majority of participants were male (78.3%), single/divorced/widowed (74.0%), aged between 18 and 29 (56.7%) and with a median age of 28 years. The predominant occupation was self-employed (30.2%) and most donors were replacement donors (71.9%).

**Table 1:** Distribution of candidate blood donors by demographic characteristics and HIV prevalence.

Characteristics	Negative n (%)	Positive n (%)	Indeterminate	Total (n) n (%)	Seroprevalence (%)
			n (%)		(95% CI)
**Overall**	3767	70	17	3854	1.8% (1.4 – 2.3%)
Gaza	2336 (62.0)	48 (68.6)	6 (35.3)	2390 (62.0)	2.0% (1.5 – 2.7%)
Inhambane	1431 (38.0)	22 (31.4)	11 (64.7)	1464 (38.0)	1.5% (0.95 – 2.3%)
**Gender**					
Female	799 (21.2)	22 (31.4)	2 (11.8)	823 (21.4)	2.8% (1.7 – 4.0%)
Male	2955 (78.4)	48 (68.6)	15 (88.2)	3018 (78.3)	1.6% (1.2 – 2.1%)
Missing	13 (0.3)	0 (0)	0 (0)	13 (0.3)	–
**Age groups**					
18-29	2146 (57.0)	29 (41.4)	12 (70.6)	2187 (56.7)	1.3% (0.89 – 1.9%)
30-39	1011 (26.8)	21 (30.0)	4 (23.5)	1036 (26.9)	2.0% (1.3 – 3.1%)
40-49	458 (12.2)	15 (21.4)	0 (0.0)	473 (12.3)	3.2% (1.8 – 5.2%)
50-59	122 (3.2)	5 (7.1)	1 (5.9)	128 (3.2)	3.9% (1.3 – 8.9%)
60-65	25 (0.7)	0 (0.0)	0 (0.0)	25 (0.6)	0% (0 – 13.7%)
Missing	5 (0.1)	0 (0.0)	0 (0.0)	5 (0.1)	–
**Marital Status**					
Married	961 (25.5)	27 (38.6)	1 (5.9)	989 (25.7)	2.7% (1.8 – 4.0%)
Single / Divorced / Widow	2792 (74.1)	43 (61.4)	16 (94.1)	2851 (74.0)	1.5% (1.1 – 2.0%)
Missing	14 (0.4)	0 (0.0)	0 (0.0)	14 (0.4)	–
**Formal Education**					
None	201 (5.3)	9 (12.9)	0 (0.0)	210 (5.4)	4.3% (2.0 – 8.0%)
Primary/Secondary /Technical	3218 (85.4)	59(84.3)	16 (94.1)	3293 (85.4)	1.8% (1.4 – 2.3%)
Higher	272 (7.2)	1 (1.4)	1 (5.9)	274 (7.1)	0.4% (0.01 – 2.0%)
Refused to answer	62 (1.6)	1 (1.4)	0 (0.0)	63 (1.6)	1.6% (0.04 – 8.5%)
Missing	14 (0.4)	0 (0.0)	0 (0.0)	14 (0.4)	–
**Occupation**					
Unemployed	546 (14.5)	13 (18.6)	3 (17.6)	562 (14.6)	2.3% (1.2 – 3.9%)
Self employed	1125 (29.8)	34 (48.6)	5 (29.4)	1164 (30.2)	2.9% (2.0 – 4.1%)
Student only	944 (25.1)	10 (14.3)	4 (23.5)	958 (25.0)	1.0% (0.5 – 1.9%)
Private agent	407 (10.8)	7 (10.0)	2 (11.8)	416 (10.8)	1.7% (0.7 – 3.5%)
Public agent	729 (19.4)	6 (8.6)	3 (17.6)	738 (19.1)	0.8% (0.3 – 1.8%)
Missing	16 (0.4)	0 (0.0)	0 (0.0)	16 (0,4)	–
**Donation type**					
Replacement	2706 (71.8)	55 (78.6)	10 (58.8)	2771 (71.9)	2.0% (1.5 – 2.6%)
First donation voluntary	483 (12.8)	10 (14.3)	3 (17.6)	496 (12.9)	2.0% (1.0 – 3.7%)
Regular voluntary	564 (15.0)	5 (7.1)	4 (23.5)	573 (14.9)	0.9% (0.3 – 2.0%)
Missing	14 (0.4)	0 (0.0)	0 (0.0)	14 (0.4)	–

The seroprevalence of HIV among study participants was 2.0% (48 infections; 95% Confidence Interval (CI): 1.5-2.7, Table 1) in Gaza province and 1.5% (22; 95% CI: 0.95-2.3) in Inhambane province. Higher seroprevalence was observed among females, 2.8% (22; 95% CI: 1.7-4.0) and married individuals, 2.7% (27; 95% CI 1.8-4.0). Replacement donors and first-time volunteers had a higher prevalence with 2.0% (55; 95% CI 1.5-2.6) and 2.0% (10; 95% CI: 1.0-3.7), respectively, compared to regular voluntary donors 0.9% (5; 95% CI: 0.3-2.0).

### Performance of the routine testing algorithm

The sensitivity and specificity of the current routine testing algorithm, Determine HIV 1/2 + Uni-Gold HIV, compared to the Merilisa HIV Gen 4 + Uni-Gold HIV (Flow diagram of study participants tested by RDTs and ELISA can be found in the supplementary material- S1 Fig to S3 Fig) were 85.5% (95% CI: 74.2 - 93.1%, Table 2) and 99.6% (95% CI: 99.3-99.8), respectively. Positive and negative agreements were 79.9% (95% CI: 66.2%-87.1%) and 99.8% (95% CI: 99.5%-99.9%), respectively, with Cohen’s kappa of 0.81 (0.62-1.01).

**Table 2:** Technical performance of rapid routine tests and new rapid HIV tests compared to the Merilisa HIV Gen 4.

Assay	Merilisa HIV Gen 4 + Unigold					Diagnostic measures (95%CI)		Agreement measures (95% CI)		
	TP	FN	FP	TN	Total	Sensitivity	Specificity	Positive	Negative	Cohen’s Kappa
**Determine HIV 1/2 + Uni-Gold HIV**	53	9	15	3695	3772	85.5% (74.2%-93.1%)	99.6% (99.3%-99.8%)	79.9% (66.2%-87.1%)	99.8% (99.5%-99.9%)	0.8 (0.62-1.0)
**Determine HIV Early Detect**	55	7	20	3705	3787	88.7% (78.1%-95.3%)	99.5% (99.2%-99.7%)	73.3% (61.9%-82.9%)	99.8% (99.6%-99.9%)	0.8 (0.62-0.98)
**MERISCREEN HIV1–2 WB test**	54	6	13	3375	3448	90.0% (79.5%-96.2%)	99.6% (99.3%-99.8%)	80.6% (69.1%-89.2%)	99.8% (99.6%-99.9%)	0.8 (0.67-1.0)

TP (True Positive): rapid test positive, reference standard positive; FP (False Positive): rapid test positive, reference standard negative; FN (False Negative): rapid test negative, reference standard positive; and TN (True Negative): rapid test negative, reference standard negative.

When assessing the Determine HIV 1/2 + Uni-Gold HIV against the molecular assay cobas MPX (Flow diagram of study participants tested by Serological tests and cobas MPX can be found in the supplementary material- S4 Fig to S7 Fig), the sensitivity was lower at 64.0% (95% CI: 49.2% -77.1%, Table 3) and the specificity was 99.1% (95% CI: 98.6%-99.4%). Positive and negative agreements were 56.1% (95% CI: 42.4%-69.3%) and 99.3% (95% CI: 98.9%-99.6%), respectively, with a Cohen’s kappa of 0.59 (95% CI: 0.28-0.90).

**Table 3:** Technical performance of HIV rapid tests, Merilisa HIV Gen 4, compared to the cobas MPX (molecular test).

Assay	Cobas MPX					Diagnostic measures (95%CI)		Agreement measures (95% CI)		
	TP	FN	FP	TN	Total	Sensitivity	Specificity	Positive	Negative	Cohen’s Kappa
**Merilisa HIV Gen 4 + Uni-Gold**	33	17	15	2641	2706	66.0% (51.2%-78.8%)	99.4% (99.1%-99.7%)	68.8% (53.7%-81.3%)	99.4% (99.0%-99.6%)	0.7 (0.33-0.99)
**Determine HIV 1/2 + Uni-Gold HIV**	32	18	25	2628	2703	64.0% (49.2% -77.1%)	99.1% (98.6%-99.4%)	56.1% (42.4%-69.3%)	99.3% (98.9%-99.6%)	0.6 (0.28-0.89)
**Determine HIV Early Detect**	35	16	25	2639	2715	68.6% (54.1% - 80.9%)	99.1% (98.6 - 99.4%)	58.3% (44.9%-70.9%)	99.4% (99.0%-99.7%)	0.6 (0.349-0.897)
**Meriscreen HIV1–2 WB test**	34	14	24	2437	2509	70.8% (55.9%-83.0%)	99.0% (98.6%-99.4%)	58.6% (44.9%-71.4%)	99.4% (99.0%-99.7%)	0.6 (0.36-0.91)

TP (True Positive): serological test, reference standard positive; FP (False Positive): serological test positive, reference standard negative; FN (False Negative): serological test negative, reference standard positive; and TN (True Negative): serological test negative, reference standard negative.

The probability of undetected positive results in the Determine HIV 1/2 + Uni--Gold HIV per 1,000 blood donors was 6.7 (95% CI: 4.0-10.7; Fig 1).

**Fig 1:**
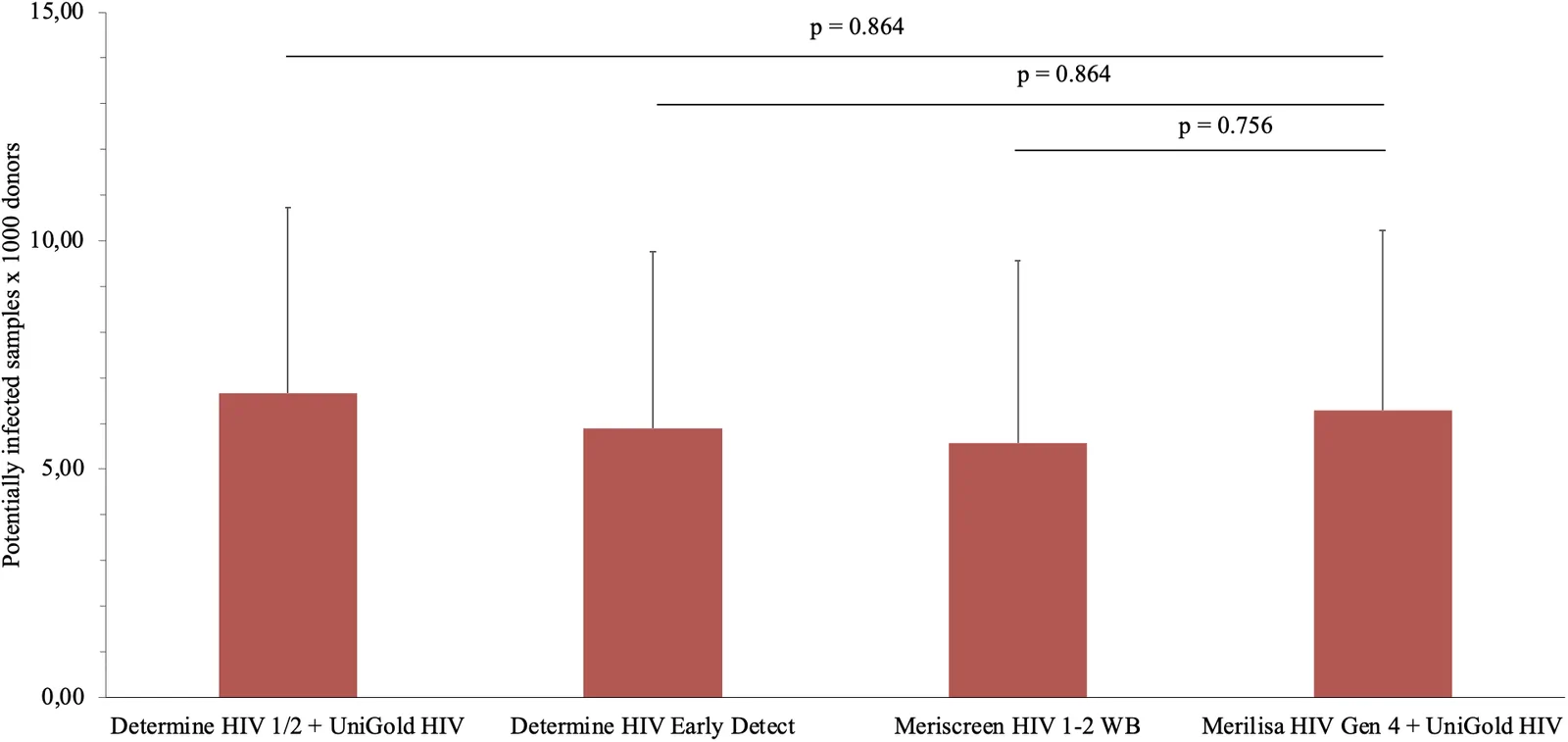
Potentially HIV-infected blood donations per test. Denominators for each estimate are as follows: Determine HIV 1/2 + Uni-Gold HIV (n = 2,703); Determine HIV Early Detect (n = 2,715); Meriscreen HIV1–2 WB (n = 2,509); Merilisa HIV Gen 4 + UniGold HIV (n = 2,706). No statistically significant differences were observed between any of the serological assays (all p‑values > 0.05).

### Performance of determine HIV early detect

Sensitivity and specificity of the Determine HIV Early Detect test, when compared to Merilisa HIV Gen 4 + Uni-Gold HIV, were 88.7% (95% CI: 78.1%-95.3%, Table 2) and 99.5% (95% CI: 99.2%-99.7%), respectively. The positive and negative agreements were 73.3% (95% CI: 61.9%-82.9%) and 99.8% (95% CI: 99.6%-99.9%), respectively. The Cohen’s kappa for this comparison was 0.80 (95% CI: 0.62-0.98).

With reference to the cobas MPX, the Determine HIV Early Detect sensitivity was 68.6% (95% CI: 54.1% - 80.9%, Table 3) and specificity was 99.1% (95% CI: 98.6% - 99.4%). The positive and negative agreements were 58.3% (95% CI: 44.9%-70.9%) and 99.4% (95% CI: 99.0%-99.7%), respectively. The Cohen’s kappa for this assessment was 0.62 (95% CI: 0.35-0.90).

The probability of undetected positive results in the Determine HIV Early Detect per 1,000 blood donors was 5.9 (95% CI: 3.5-9.8, Fig 1).

### Performance of Meriscreen HIV1–2 Western Blot

The sensitivity and specificity of the Meriscreen HIV1–2 WB test compared to Merilisa HIV Gen 4 + Uni-Gold HIV, were 90.0% (95% CI: 79.5%-96.2%, Table 2) and 99.6% (95% CI: 99.3%-99.8%), respectively. The positive and negative agreements were 80.6% (95% CI: 69.1%-89.2%) and 99.8% (95% CI: 99.6%-99.9%), and Cohen’s kappa was 0.85 (95% CI: 0.68-1.02).

The analytical performance against the cobas MPX was lower, with a sensitivity of 70.8% (95% CI: 55.9%-83.0%, Table 3) and a specificity of 99.0% (95% CI: 98.6%-99.4%). The positive and negative agreements were 58.6% (95% CI: 44.9%-71.4%) and 99.4% (95% CI: 99.0%-99.7%), respectively. The Cohen’s kappa was 0.63 (95% CI: 0.36-0.91).

The probability of undetected positive results in the Meriscreen HIV1–2 WB test per 1,000 blood donors was 5.6 (95% CI: 3.1-9.6, Fig 1).

### Performance of Merilisa HIV Gen 4

The overall sensitivity and specificity of the Merilisa HIV Gen 4 + Uni-Gold HIV compared to the cobas MPX were 66.0% (95% CI: 51.2%-78.8%, Table 3) and 99.4% (95% CI: 99.1%-99.7), respectively. The positive and negative agreements were 68.8% (95% CI: 53.7%-81.3%) and 99.4% (95% CI: 99.0%-99.6%), respectively, and the Cohen’s kappa was 0.67 (95% CI: 0.34-0.99).

The probability of undetected positive specimens was 6.3 (95% CI: 3.7-10.2, Fig 1) per 1,000 blood donors.

### Assessment of the potential impact of bias on HIV testing using NAT

Comparison of demographic characteristics between NAT-tested and untested samples showed similar age and sex distributions, although HIV seroprevalence was higher in the NAT-tested subgroup than in the untested subgroup (2.2% vs. 1.1%, p = 0.011).

To assess the potential impact of verification bias, we conducted a sensitivity analysis restricted to the 2,467 samples (64.0% of the total) for which results from all testing modalities were available. The results were consistent with the primary analysis, with sensitivity of serological tests compared to NAT ranging from 66.7% to 71.1% and FOR ranging from 5.3 to 6.1 per 1,000 donors (S2 Table).

## Discussion

Blood banks located in remote settings in sub-Saharan Africa urgently need point-of-care technologies for HIV and other TTIs. In recent decades, many of these settings have relied on rapid antibody assays to detect HIV infection in candidate blood donors, which may compromise the safety of blood donations.

In this study, RDTs showed good agreement (0.80) and similar specificity (99.5% to 99.6%) to ELISA-based methods. The sensitivities of the evaluated RDTs ranged from 85.5% to 90.0%, with the lowest sensitivity observed in the Determine + Uni-Gold HIV algorithm at 85.5%. Nevertheless, the sensitivities presented by the RDTs evaluated in this study fall below the World Health Organization (WHO) recommendation for screening TTIs (sensitivity > 99.0%) [3].

All types of serological assays, including RDTs, ELISA and WB, had a low sensitivity (64% to 70.8%) and moderate agreement (0.6 to 0.7) when compared to the molecular test cobas MPX. The specificity ranged from 99.0 to 99.4. These results are in line with several studies that recommend the use of molecular tests for HIV screening in blood donors [14,20,21].

Verification bias is a potential concern because not all samples underwent NAT testing due to logistical constraints and limited sample volume. HIV seroprevalence was higher among NAT-tested samples than among untested samples, suggesting some selection into NAT testing. However, the sensitivity analysis restricted to samples with complete results produced findings consistent with the primary analysis, with overlapping confidence intervals for the key performance measures. This consistency suggests that verification bias was present but did not materially change the main finding that serological tests had limited sensitivity for blood donor screening when compared with NAT.

The low sensitivity of all serological tests evaluated in this study, including assays designed to detect antigen, supports the introduction of molecular assays for pathogen screening in blood donors in developing countries. Testing strategies could include centralized processing, with samples from peripheral sites transported to urban reference laboratories equipped with high-throughput automated platforms. However, point-of-care testing will remain essential for remote settings where centralized testing may not be feasible.

Many African countries are currently using point-of-care tests for HIV and tuberculosis diagnosis and treatment monitoring in clinical settings [22,23]. In Mozambique, for example, point-of-care equipment for HIV diagnosis in exposed infants and for tuberculosis detection is available in 138 and 232 health facilities, respectively [13]. While these technologies might be fit for diagnosing TTIs in blood banks, this application has not been explored. The development of novel point-of-care molecular tests for TTIs or of new assays using currently available point-of-care equipment plays a pivotal role in blood donation safety in under-resourced settings in sub-Saharan Africa.

Despite the importance of introducing NAT in reducing transfusion risk, studies on the cost-effectiveness of its implementation for screening for HIV and other bloodborne pathogens have yielded contradictory results [24–26]. The introduction of NAT should be based on a rigorous cost-effectiveness analysis that accounts for the epidemiological situation, the capacity of the health care system, and the opportunity cost of each investment.

Improving the performance of pathogen screening tests is not the only measure to improve transfusion safety. Other interventions such as strict pre-screening, attraction, recruitment and retention of volunteer blood donors have been shown to be effective in reducing the risk of TTIs [27,28]. Pre-screening blood donors with a higher capacity to exclude candidates at risk of TTI has shown to have an impact on reducing transfusion risk [29,30].

The results of this study provide valuable insights that can inform transfusion services about the best-fitted testing algorithm and other strategies to improve transfusion safety. However, some limitations may have influenced the results obtained: First, introducing two new RDTs into routine blood bank workflows may have increased workload and affected performance of the routine Determine HIV 1/2 + Uni-Gold HIV algorithm. Second, cobas MPX testing was not performed for all samples with HIV serological results because some samples had insufficient volume or poor quality, reducing the number of samples available for comparison with NAT.

## Conclusion

The rapid tests evaluated for HIV screening had variable sensitivity and all performed below the WHO recommendation for screening blood donors. Overall, these findings indicate a substantial residual risk of HIV transmission when blood banks rely on serological testing alone. Introducing molecular testing for HIV in blood banks in developing countries could improve transfusion safety and support broader efforts to reduce HIV and other transfusion-transmissible infections as public health problems.

## Supporting information

S1 Text Questionnaire for participants. (DOCX)

S1 Table Characteristics of serological tests. (DOCX)

S1 Fig Flow diagram of study participants tested Determine HIV 1/2 + UniGold and Merilisa+Unigold. (TIF)

S2 Fig Flow diagram of study participants tested by Determine HIV Early detect and Merilisa+Unigold. (TIF)

S3 Fig Flow diagram of study participants tested by Meriscreen HIV1–2WB and Merilisa+Unigold. (TIF)

S4 Fig Flow diagram of study participants tested by Determine HIV 1/2 + UniGold and Cobas MPX. (TIF)

S5 Fig Flow diagram of study participants tested by Determine HIV Early detect and Cobas MPX. (TIF)

S6 Fig Flow diagram of study participants tested by Meriscreen HIV1–2WB and Cobas MPX. (TIF)

S7 Fig Flow diagram of study participants tested by Merilisa+Unigold and Cobas MPX. (TIF)

S2 Table Performance of rapid serological tests in complete-case samples. (DOC)

S1 Data Study data set. (XLSX)
