# Supplementary material for: High transmission risk of Human Immunodeficiency Virus in blood transfusions: A critical evaluation of blood bank serological tests in Mozambique
Source: PLOS Glob Public Health. 2026 Sep 30;6(9):e0007398. doi: 10.1371/journal.pgph.0007398 (PMC13626358; doi:10.1371/journal.pgph.0007398)
Supplement: Supplementary file 1 [file pgph.0007398.s001.docx]

**MINISTÉRIO DA SAÚDE**

**Instituto Nacional de Saúde de Moçambique**

# Departamento de Imunologia

## **“**Impacto de uso de novos testes rápidos de diagnóstico de HIV, HBV, HCV e *Treponema pallidum* na segurança transfusional em dadores de sangue em Gaza e Inhambane**”**

Dados sócio-demográficos

| Número do Dador: \|___\|___\|___\|___\|___\|___\|___\|___\|___\|  Número de Frasco  \|___\|___\|___\|___\|___\|___\|___\|___\|___\| | Idade: \|___\|___\| anos  Tipo de Dador:  1.Repositor  2. Voluntário de primeira doação  3.Voluntário de repetição |
| --- | --- |
| Género:  1. Masculino:  2. Feminino: | Grau de Escolaridade:  1. Analfabeto:  2. Primário:  3. Secundário / Técnico :  4. Superior: |
| Estado civil:  1. Solteiro/a:  2. Casado/a:  3. Divorciado/a:  4. Viúvo/a: | Cor da pele:  1. Negra:  2. Mista:  3. Branca: |
| Local de nascimento:  Local de Residência (província): | Profissão: |

Observações do entrevistador: _______________________________________________________________________________________________________________________________________________________________________________________________________________________________________________________________

Gaza/Inhambane, _________ (Dia) de ___________________(Mês) de_______(ano)

_________________________________________________

(Assinatura do Dador)

_________________________________________________

(Assinatura do entrevistador)

**MINISTRY OF HEALTH**

**Instituto Nacional de Saúde of Mozambique**

# Departamento de Imunologia

“Impact of the use of new rapid diagnostic tests for HIV, HBV, HCV, and Treponema pallidum on transfusion safety in blood donors in Gaza and Inhambane”

Socio-Demographic data

| Donor number: \|___\|___\|___\|___\|___\|___\|___\|___\|___\|  Tube number  \|___\|___\|___\|___\|___\|___\|___\|___\|___\| | Age: \|___\|___\| years  Donation type:  1.Replacement  2. First-time voluntary  3.Regular voluntary |
| --- | --- |
| Gender:  1. Male:  2. Female: | Education:  1. None:  2. Primary:  3. Secondary:  4. Higher: |
| Marital Status:  1. Single:  2. Married:  3. Divorced:  4. Widow: | Skin color:  1. Black:  2. Mixed:  3. White: |
| Place of birth:  Place of residence (provínce): | Occupation: |

Interviewer’s observation: ______________________________________________________________________________________________________________________________________________________________________________________________________________________________________________________________

Gaza/Inhambane, _________ (Day) of ___________________ (Month) _________year

_________________________________________________

(Donor Signature)

_________________________________________________

(Interviewer’s Signature)
