# Supplementary material for: High transmission risk of Human Immunodeficiency Virus in blood transfusions: A critical evaluation of blood bank serological tests in Mozambique
Source: PLOS Glob Public Health. 2026 Sep 30;6(9):e0007398. doi: 10.1371/journal.pgph.0007398 (PMC13626358; doi:10.1371/journal.pgph.0007398)
Supplement: Supplementary file 2 [file pgph.0007398.s002.docx]

**S1 Table Characteristics of serological tests**

|  | **Determine HIV 1/2** | **UniGold HIV** | **Determine HIV Early Detect** | **MERISCREEN HIV1-2 WB test** | **Merilisa HIV Gen 4** |
| --- | --- | --- | --- | --- | --- |
| **Type of assay** | Lateral flow immunochromatographic assay | Lateral flow  Immunochromatographic assay | Lateral flow immunochromatographic assay | Lateral flow  Immunochromatography assay | Enzyme immunoassay |
| **Sample** | Serum, Plasma, Whole Blood | Serum, Plasma, Whole Blood | Serum, Plasma, Whole Blood | Serum, Plasma, Whole Blood | Serum, Plasma, |
| **Volume of sample** | 50µl | 60µl | 50µl | 10µl | 100µl |
| **Time** | 15 minutes | 10 minutes | 20 minutes | 20 minutes | 120 minutes |
| **Equipment needed** | Timer | Timer | Timer | Timer | Timer, Incubator, Plate Washer, Plate Reader, Pipette |
| **Target** | Antibodies to HIV-1 and HIV-2 | Antibodies to HIV-1 and HIV-2 | HIV-1 p24 antigen  Antibodies to HIV-1 and HIV-2 | Antibodies (IgG, IgA, and IgM) specific to HIV-1 and HIV-2 | HIV p24 antigen  Antibodies to HIV-1 and HIV-2 |
| **Results interpretation** | Reactive  Non-Reactive  Invalid | Reactive  Non-Reactive  Invalid | Reactive  Ag Reactive  Ab Reactive  Ag+Ab Reactive  Invalid | Negative  HIV 1 Positive  HIV 2 Positive  HIV 1 and 2 Positive  Invalid | Negative  Positive  Undetermined |
| **Sensitivity and specificity (according to the manufacturer)** | 100% sensitivity  99.75% specificity | 100% sensitivity  100% specificity | 100% sensitivity  99.72% specificity | 100% sensitivity  100% specificity | 100% sensitivity  99.5% specificity |
