# Supplementary material for: High transmission risk of Human Immunodeficiency Virus in blood transfusions: A critical evaluation of blood bank serological tests in Mozambique
Source: PLOS Glob Public Health. 2026 Sep 30;6(9):e0007398. doi: 10.1371/journal.pgph.0007398 (PMC13626358; doi:10.1371/journal.pgph.0007398)
Supplement: Supplementary file 3 [file pgph.0007398.s010.doc]

**Technical performance of rapid routine tests and new rapid HIV tests compared to the Merilisa HIV Gen 4***

| **Assay** | **Merilisa HIV Gen 4 + Unigold** | | | | | | **Diagnostic measures (95%CI)** | | **Agreement measures (95% CI)** | | |
| --- | --- | --- | --- | --- | --- | --- | --- | --- | --- | --- | --- |
|  | True positive | | False Negative | False Positive | True Negative | Total | Sensitivity | Specificity | Positive | Negative | Cohen's Kappa |
| **Determine HIV ½ + Unigold HIV** | | 43 | 5 | 13 | 2423 | 2484 | 89.6%  (77.3%-96.5%) | 99.5%  (99.1%-99.7%) | 76.8%  (63.6%-87.0%) | 99.8%  (99.5%-99.9%) | 0.82  (0.63-1.02) |
| **Determine HIV Early Detect** | | 45 | 3 | 10 | 2426 | 2484 | 93.8%  (82.8%-98.7%) | 99.6%  (99.2%-99.8%) | 81.8%  (69.1%-90.9%) | 99.9%  (99.6%-100.0%) | 0.87  (0.70-1.04) |
| **MERISCREEN HIV1-2 WB Test** | | 45 | 3 | 10 | 2426 | 2484 | 93.8%  (82.8%-98.7%) | 99.6%  (99.2%-99.8%) | 81.8%  (69.1%-90.9%) | 99.9%  (99.6%-100.0%) | 0.87  (0.70-1.04) |

***** Only participants with complete data for all tests were included in the analysis

TP (True Positive): rapid test positive, reference standard positive; FP (False Positive): rapid test positive, reference standard negative; FN (False Negative): rapid test negative, reference standard positive; and TN (True Negative): rapid test negative, reference standard negative.

**Technical performance of HIV rapid tests, Merilisa HIV Gen 4, compared to the Cobas MPX (molecular test) *^1^**

| **Assay** | **Cobas MPX** | | | | | | **Diagnostic measures (95%CI)** | | **Agreement measures (95% CI)** | | |
| --- | --- | --- | --- | --- | --- | --- | --- | --- | --- | --- | --- |
|  | TP | | FN | FP | TN | Total | Sensitivity | Specificity | Positive | Negative | Cohen's Kappa |
| **Merilisa HIV Gen 4 + Unigold** | | 33 | 13 | 15 | 2423 | 2484 | 71.7%  (56.5%-84.0%) | 99.4%  (99.0%-99.7%) | 68.8%  (53.7%-81.3%) | 99.5%  (99.1%-99.7%) | 0.70  (0.38-1.01) |
| **Determine HIV ½ + Unigold HIV** | | 31 | 15 | 25 | 2413 | 2484 | 67.4%  (52.0%-80.5%) | 99.0%  (98.5%-99.3%) | 55.4%  (41.5% -68.7%) | 99.4%  (99.0%-99.7%) | 0.60  (0.29-0.91) |
| **Determine HIV Early Detect** | | 33 | 13 | 22 | 2416 | 2484 | 71.7%  (56.5%-84.0%) | 99.1%  (98.6%-99.4%) | 60.0%  (45.9%-73.0%) | 99.5%  (99.1%-99.7%) | 0.65  (0.35-0.94) |
| **MERISCREEN HIV1-2 WB Test** | | 33 | 13 | 22 | 2416 | 2484 | 71.7%  (56.5%-84.0%) | 99.1%  (98.6%-99.4%) | 60.0%  (45.9%-73.0%) | 99.5%  (99.1%-99.7%) | 0.65  (0.35-0.94) |

*^1^ Only participants with complete data for all tests were included in the analysis

TP (True Positive): serological test, reference standard positive; FP (False Positive): serological test positive, reference standard negative; FN (False Negative): serological test negative, reference standard positive; and TN (True Negative): serological test negative, reference standard negative.
